# The Prevalence of Fatigue Following Deep Brain Stimulation Surgery in Parkinson's Disease and Association with Quality of Life

**DOI:** 10.1155/2012/769506

**Published:** 2012-05-13

**Authors:** Benzi M. Kluger, Veronica Parra, Charles Jacobson, Cynthia W. Garvan, Ramon L. Rodriguez, Hubert H. Fernandez, Amanda Fogel, Barry M. Skoblar, Dawn Bowers, Michael S. Okun

**Affiliations:** ^1^Department of Neurology, University of Colorado Denver, P.O. Box 6511, Aurora, CO 80045, USA; ^2^Department of Neurology, University of Florida, Gainesville, FL 32610, USA; ^3^Department of Educational Psychology, University of Florida, Gainesville, FL 32611, USA; ^4^Department of Clinical and Health Psychology, University of Florida, Gainesville, FL 32610, USA

## Abstract

Fatigue is a common and disabling nonmotor symptom seen in Parkinson's disease (PD). While deep brain stimulation surgery (DBS) improves motor symptoms, it has also been associated with non-motor side effects. To date no study has utilized standardized instruments to evaluate fatigue following DBS surgery. Our objective was to determine the prevalence of fatigue following DBS surgery in PD its impact on quality of life and explore predictive factors. We recruited 44 PD subjects. At least one year following DBS placement, we administered the Fatigue Severity Scale (FSS), the Parkinson's Disease Questionnaire (PDQ-39), the Beck Depression Inventory, the Beck Anxiety Inventory, the UPDRS, and a neuropsychological battery. Fifty-eight percent of subjects had moderate to severe fatigue. Fatigue was significantly associated with quality of life, depression, and anxiety. Depression preoperatively was the only predictive factor of fatigue. Fatigue is common following DBS surgery and significantly impacts quality of life.

## 1. Introduction

 Fatigue is one of the most common and disabling non-motor symptoms seen in Parkinson's disease (PD) with studies showing a prevalence of moderate to severe fatigue in 33–58% of PD patients [1]. Fatigue is reported by one-third of patients as their most disabling symptom and is associated with a worse quality of life [2, 3]. The cause of fatigue in PD remains unclear. Fatigue is seen even in newly diagnosed PD patients and does not correlate with motor symptoms, other measures of disease severity, sleep dysfunction, or medication use [1–4]. While studies have shown depression to be the variable most strongly and consistently associated with fatigue in PD they also demonstrate that these symptoms are frequently independent [5, 6]. Cognitive dysfunction is also associated with fatigue in PD in some studies but is clearly independent from fatigue in many patients [4, 7].

 While deep brain stimulation surgery (DBS) has proven to be an effective treatment to address the motor symptoms/fluctuations in PD, more recent literature has demonstrated that DBS may also be associated with significant non-motor side effects, including changes in speech, worsening cognition, and negative mood states [8–11]. These non-motor side effects may limit the benefit of DBS for some patients, particularly in terms of overall quality of life (QOL). Fatigue has been largely unexamined in this population, but given the prevalence of other non-motor issues in this population, the potential for adverse effects on QOL is worthy of further study. Funkiewiez and colleagues utilized the Addiction Research Center Inventory (ARCI) questionnaire Parkinson's Disease to evaluate fatigue on and off of DBS patients three months following surgery [12]. They observed some improvement in momentary “fatigue” when subjects were in their on-DBS state. However, it is unclear how their measure of fatigue, designed to test the affect of psychoactive substances, relates to fatigue in PD, and whether the acute on/off effects are representative of the chronic effects of long-term DBS stimulation. 

 To date, no study has utilized standardized fatigue instruments to evaluate the prevalence of fatigue or its impact on HR QOL following DBS surgery. Our primary objectives were to (1) determine the prevalence of clinically significant fatigue in PD patients who have undergone DBS surgery; (2) determine the association of fatigue with health related quality of Life (HR-QOL). We hypothesized that fatigue is common following DBS and inversely associated with HR-QOL. Exploratory aims were to (1) identify disease characteristics, mood, or neuropsychological variables associated with fatigue in this cohort; (2) identify whether any aspects or preoperative testing predicted fatigue following DBS. We hypothesized that depression and cognitive dysfunction would be associated with fatigue in this population and also predict fatigue following DBS when detected preoperatively.

## 2. Methods

The investigational protocol was approved by the University of Florida Institutional Review Board. All participants gave written informed consent.

### 2.1. Participants

Forty-four consecutive patients with PD who were at least one year after DBS surgery, able to give consent, and interested in participating in research were recruited from the University of Florida Movement Disorder Clinics and clinical research database from May 2006 to July 2007.

### 2.2. Procedure

All subjects completed the following questionnaires at the time of recruitment: the Fatigue Severity Scale (FSS) [13], the Parkinson's Disease Questionnaire Quality of Life-39 (PDQ-39) [14], the Apathy Scale [15], the Beck Anxiety Inventory (BAI) [16], and the Beck Depression Inventory (BDI-II) [17]. These scales have all been validated within the PD population [14, 18–21]. Moderate to severe fatigue was defined as a score of 4 or greater on the FSS. Disease severity was assessed using the Hoehn and Yahr scale [22] and motor section of the Unified Parkinson's Disease Rating Scale (UPDRS) [23] in the on-DBS and On medication state.

For our exploratory objectives, a substantial subset of patients (*N* = 28) had additional data available through our clinical research database collected within six months of recruitment for the current study and also pre-operatively. This data included the State Trait Anxiety Inventory [24], and a standard battery of neuropsychological function: Mini Mental Status Exam [25], Dementia Rating Scale-2 [26], Weschler Abbreviated Scale of Intelligence [27], Wechsler Adult Intelligence Scale III (test the digit span, and digit symbol) [28], Stroop Color and Word Test [29], Trail A and B [30], Boston Naming test [31], Controlled Oral Word Association Test [32], Category Fluency Test [33], Hopkins Verbal Learning Test-Revised [34], Weschler Memory Scale III [28], Judgment of Line Orientation Test [35], Face Recognition Test [36], and Benton Visual Retention Test [37]. Disease severity was assessed preoperatively (on medications) using the Hoehn and Yahr scale [22] and the motor section of the UPDRS [23].

### 2.3. Statistical Analysis

Statistical analysis was performed using SAS version 9.2 (SAS Inc., Cary, NC). Wilcoxon rank-sum test was used to determine differences between groups. Spearman's correlation was performed to determine relationships between continuous variables. Chi-square test, or Fisher exact test, was when appropriate used to assess the relationship between categorical variables. Linear regression models were used to assess the association of multiple variables, including controlling for confounding variables. *P* values <.05 were considered significant.

## 3. Results

### 3.1. Prevalence of Fatigue and Characteristics of the High and Low Fatigue Patients

Fifty-eight percent of subjects (95% confidence interval: 43–73%) had a score of 4 or greater on the FSS. The mean FSS score for this cohort was 4.2 (SD 1.3). When subjects were divided into low (FSS < 4; mean FSS 3.0, SD 0.6) and high (FSS ≥ 4; mean FSS 5.2, SD 0.8) fatigue groups, significant differences were found only in anxiety (BAI 8.0 versus 15.9, *P* = 0.001) and quality of life (PDQ 39 23.8 versus 33.0, *P* = 0.02) in low versus high fatigue groups, respectively. Trends for differences were also noted in depression (BDI 7.4 versus 12.0, *P* = 0.07) and disease duration (17.1 versus 14.1 yrs, *P* = 0.10).  Table 1 outlines these comparisons and summarizes group demographic data.

### 3.2. Correlation of Fatigue with Quality of Life

FSS scores were significantly associated with the total score of the PDQ 39 (*r* = 0.37, *P* = 0.01) as well as the majority of PDQ subscales including mobility, emotional well-being, social support, cognition, and bodily discomfort. See Table 2 for the correlation of fatigue with PDQ 39 subscores. Linear regression models demonstrated that the association between the FSS and PDQ 39 remained significant even after controlling for age, disease duration, and motor severity (FSS F-value in model 9.83, *P* < 0.01).

### 3.3. Correlation of Fatigue with Disease Severity, Mood, and Neuropsychological Test Results

Fatigue was associated with depression as measured by the BDI (*r* = 0.47, *P* = 0.01) and anxiety as measured by the BAI (*r* = 0.49, *P* < 0.01) but not with apathy, age, disease duration, time since DBS, the UPDRS (on or off), Hoehn and Yahr (on or off), anxiety measured with State and Trait Anxiety Inventory (STAI), or any neuropsychological test scores.

### 3.4. Factors Predictive of Fatigue in PD Patients Who Have Undergone DBS Surgery

Depression as measured by BDI was the only preoperative measure associated with fatigue (Spearman's *r* = 0.48, *P* = 0.03). While there was a strong correlation between pre and postoperative BDI scores (*r* = 0.68, *P* < 0.01), preoperative BDI scores remained associated with the FSS even when controlled for postoperative BDI in a linear regression model (BDI F-value in model 4.64, *P* = 0.05). There was no association between DBS target and fatigue (see Table 1; Fisher exact test *P* > 0.05), anxiety (as measured by STAI only), apathy, or any neuropsychological test scores.

## 4. Discussion

Fatigue is a common non-motor symptom following DBS surgery and seems to significantly impact HR-QOL. Our finding of 58% prevalence among DBS subjects is on the higher end of the range of previously reported fatigue prevalence in PD subjects without DBS which have ranged from 33–58% [1]. Our data suggest that fatigue may be related to mood disturbances, and this finding may have implications for future research into the causes of fatigue in DBS subjects.

 The association of depression and fatigue has been well documented in PD patients [1, 38]. The correlation with anxiety as measured by the BAI, however, has not been reported. Given the high prevalence of both symptoms, it is not surprising that there would be some comorbidity between these two symptoms [39]. It should be noted, however, that the State and Trait Anxiety Inventory failed to show a significant correlation with fatigue. This finding suggested that part of this association may be related to test factors, for instance the weight of somatic questions on the BAI. It is also possible that certain types of anxiety disorders (e.g., panic disorders which is better screened by the BAI) may be more associated with fatigue and better measured by certain scales [40]. Whether this association has relevance to PD patients without DBS is unknown but worthy of further study given the prevalence of both fatigue and anxiety in PD.

 The lack of correlation between fatigue and apathy or neuropsychological performance is notable. One prior investigation demonstrated a trend toward correlation between fatigue and the Wisconsin Card Sorting task as well as a significant association with frontal hypometabolism on PET [41]. A second study suggested that MMSE scores were higher in subjects with greater fatigue, but this result may have been confounded by other differences between high and low MMSE groups [7]. Our findings may suggest that fatigue arises independently of other neuropsychological symptoms in patients following DBS. However, objective testing specifically for mental fatigue may prove to be more sensitive in investigating this possible association [42]. It should also be noted that DBS patients are a highly selected population to begin with and may not have sufficient variability in cognitive measures to fully explore a potential association with fatigue. Moreover, our sample size of subjects with adequate neuropsychological results (*N* = 28) is not sufficient to detect subtle associations, and testing was often not performed at the time of other subjective measurements but included if performed within a six month window.

 This study has several notable limitations, including its relatively small size, lack of longitudinal data, capture of several variables at disparate time points (e.g., neuropsychological test results), and lack of a control group. Regarding our definition of fatigue, while the FSS has been widely used to define clinically significant fatigue in many diseases, including in PD, there are clear differences between the various subjective scales and even more so with objective measures of fatigability [19, 42]. Importantly, as fatigue was not assessed prior to DBS surgery, we cannot determine whether DBS impacts fatigue in this population. Future investigations should consider using a larger sample size, assessing fatigue and other explanatory variables longitudinally (particularly prior to DBS), assessing sleep, and having a control group of PD patients on medical treatment.

### 4.1. Conclusions

Fatigue is common following DBS surgery in PD and has a negative impact on HR QOL of the patients. Fatigue in this population appears associated with depression and possibly anxiety but did not correlate with other neuropsychological tests or disease characteristics. Future research should investigate fatigue before and after DBS to determine whether DBS impacts this important symptom.

## Figures and Tables

**Table 1:** Comparison of clinical characteristics in high and low fatigue patients. Data reported as mean (standard deviation).

	Total (*n* = 44)	Low fatigue (FSS < 4) (*n* = 19)	High fatigue (FSS ≥ 4) (*n* = 25)	*P* value
Age (years)	63.3 (10.0)	61.5 (11.4)	64.7 (8.8)	0.32
Gender (% male)	86%	84%	88%	0.71*
DBS target	STN (30)	STN (14)	STN (16)	
	GPI (10)	GPI (3)	GPI (7)	0.59**
	VIM (4)	VIM (2)	VIM (2)	
Disease duration (years)	15.5 (5.5)	17.1 (6.0)	14.1 (11.8)	0.10
Months after DBS	26.1 (20.9)	27.3 (25.8)	25.3 (12.4)	0.81
Hoehn and Yahr (on)	2.5 (0.7)	2.5 (0.5)	2.5 (0.7)	0.92
UPDRS motor (on)	28.1 (12.6)	26.9 (12.7)	28.9 (12.7)	0.64
FSS	4.2 (0.8)	3.1 (0.6)	5.1 (0.8)	<0.0001
MMSE	27.8 (2.4)	28.2 (1.9)	27.2 (3.0)	0.25
BDI	9.2 (7.2)	7.4 (6.6)	12.0 (7.4)	0.07
BAI	12.2 (8.2)	8.0 (5.2)	15.9 (8.6)	**0.001**
Apathy Scale	13.2 (6.6)	11.7 (7.1)	14.4 (6.1)	0.20
PDQ 39	28.7 (13.9)	23.8 (12.0)	33.0 (14.2)	**0.02 **

Wilcoxon test was used except for: *Chi Square test was used and **Fisher's Exact Test was used.

BAI: Beck Anxiety Inventory; BDI: Beck Depression Inventory; DBS: deep brain stimulation; FSS: Fatigue Severity Scale; GPI: globus pallidus interna; MMSE: Mini Mental State Examination; PDQ: Parkinson's Disease Questionnaire; STN: subthalamic nucleus; UPDRS: Unified Parkinson's Disease Rating Scale; VIM: ventral intermediate nucleus of thalamus.

**Table 2:** Correlation of Fatigue Severity Scale with Parkinson Disease Questionnaire (PDQ) 39 subscores.

PDQ 39 subscore	Spearman's correlation (*P* value)
Total	0.37 (0.02)*
Mobility	0.33 (0.04)*
Activities of daily living	0.27 (0.08)
Emotional well-being	0.38 (0.01)*
Stigma	0.16 (0.32)
Social support	0.20 (0.20)
Cognition	0.42 (0.006)**
Communication	0.11 (0.50)
Bodily discomfort	0.49 (0.001)**

*P value significant <0.05; **P value < 0.01.

## References

[1] Friedman JH, Brown RG, Comella C (2007). Fatigue in Parkinson’s disease: a review. *Movement Disorders*.

[2] Friedman J, Friedman H (1993). Fatigue in Parkinson’s disease. *Neurology*.

[3] Van Hilten JJ, Weggeman M, Van der Velde EA, Kerkhof GA, Van Dijk JG, Roos RAC (1993). Sleep, excessive daytime sleepiness and fatigue in Parkinson’s disease. *Journal of Neural Transmission - Parkinson’s Disease and Dementia Section*.

[4] Schifitto G, Friedman JH, Oakes D (2008). Fatigue in levodopa-naïve subjects with Parkinson disease. *Neurology*.

[5] Havlikova E, Rosenberger J, Nagyova I (2008). Clinical and psychosocial factors associated with fatigue in patients with Parkinson’s disease. *Parkinsonism and Related Disorders*.

[6] Karlsen K, Larsen JP, Tandberg E, Jorgensen K (1999). Fatigue in patients with Parkinson's disease. *Movement Disorders*.

[7] Alves G, Wentzel-Larsen T, Larsen JP (2004). Is fatigue an independent and persistent symptom in patients with Parkinson disease?. *Neurology*.

[8] Kluger BM, Klepitskaya O, Okun MS (2009). Surgical Treatment of Movement Disorders. *Neurologic Clinics*.

[9] Lilleeng B, Dietrichs E (2008). Unmasking psychiatric symptoms after STN deep brain stimulation in Parkinson's disease. *Acta Neurologica Scandinavica*.

[10] Smeding HMM, Speelman JD, Koning-Haanstra M (2006). Neuropsychological effects of bilateral STN stimulation in Parkinson disease: a controlled study. *Neurology*.

[11] Witt K, Daniels C, Reiff J (2008). Neuropsychological and psychiatric changes after deep brain stimulation for Parkinson’s disease: a randomised, multicentre study. *The Lancet Neurology*.

[12] Funkiewiez A, Ardouin C, Cools R (2006). Effects of Levodopa and subthalamic nucleus stimulation on cognitive and affective functioning in Parkinson’s disease. *Movement Disorders*.

[13] Krupp LB, LaRocca NG, Muir-Nash J, Steinberg AD (1989). The fatigue severity scale. Application to patients with multiple sclerosis and systemic lupus erythematosus. *Archives of Neurology*.

[14] Peto V, Jenkinson C, Fitzpatrick R, Greenhall R (1995). The development and validation of a short measure of functioning and well being for individuals with Parkinson’s disease. *Quality of Life Research*.

[15] Starkstein SE, Mayberg HS, Preziosi TJ, Andrezejewski P, Leiguarda R, Robinson RG (1992). Reliability, validity, and clinical correlates of apathy in Parkinson’s disease. *Journal of Neuropsychiatry and Clinical Neurosciences*.

[16] Beck AT, Epstein N, Brown G, Steer RA (1988). An inventory for measuring clinical anxiety: psychometric properties. *Journal of Consulting and Clinical Psychology*.

[17] Beck AT, Steer RA, Ball R, Ranieri WF (1996). Comparison of Beck depression inventories -IA and -II in psychiatric outpatients. *Journal of Personality Assessment*.

[18] Drijgers RL, Dujardin K, Reijnders JSAM, Defebvre L, Leentjens AFG (2010). Validation of diagnostic criteria for apathy in Parkinson’s disease. *Parkinsonism and Related Disorders*.

[19] Friedman JH, Alves G, Hagell P (2010). Fatigue rating scales critique and recommendations by the Movement Disorders Society Task Force on rating scales for Parkinson’s disease. *Movement Disorders*.

[20] Leentjens AF, Dujardin K, Marsh L, Richard IH, Starkstein SE, Martinez-Martin P (2011). Anxiety rating scales in Parkinson’s disease: a validation study of the Hamilton anxiety rating scale, the Beck anxiety inventory, and the hospital anxiety and depression scale. *Movement Disorders*.

[21] Leentjens AF, Verhey FR, Luijckx GJ, Troost J (2000). The validity of the Beck Depression Inventory as a screening and diagnostic instrument for depression in patients with Parkinson's disease. *Movement Disorders*.

[22] Hoehn MM, Yahr MD (1967). Parkinsonism: onset, progression and mortality.. *Neurology*.

[23] Fahn S, Committee MotUD, Fahn S, Calne DB, Goldstein M (1987). Unified Parkinson disease rating scale. *Recent Developments in Parkinson’s Disease*.

[24] Spielberger CD, Lushene RD, Vagg PR, Jacobs GA (1983). *Manual for the State-Trait Anxiety Inventory (FormY Self-Evaluation Questionnaire)*.

[25] Folstein MF, Folstein SE, McHugh PR (1975). “Mini-mental state”. A practical method for grading the cognitive state of patients for the clinician. *Journal of Psychiatric Research*.

[26] Mattis S (2002). *Dementia Rating Scale—2 (DRS-2)*.

[27] Wechsler D (1999). *Wechsler Abbreviated Scale of Intelligence (WASI) Manual*.

[28] Weschler D (1997). *Weschler Adult Intelligence Scale*.

[29] Golden C (1978). *Stroop Color and Word Test*.

[30] Reitan R (1993). *The Halstead-Reitan Neuropsychological Test Battery: Theory and Clinical Interpretation*.

[31] Kaplan EF, Weintraub S (1983). *The Boston Naming Test*.

[32] Thurstone L (1938). *Primary Mental Abilities: Psychometric Monographs*.

[33] Goodglass H (1972). *Assessment of Aphasia and Related Disorders*.

[34] Brant J (2001). *Hopkins Verbal Learning Test*.

[35] Benton AL, Varney NR, DeS. Hamsher K. K (1978). Visuospatial judgment. A clinical test. *Archives of Neurology*.

[36] Benton AL, Hamsher K, Varney N, Spreen O (1994). *Contributions to Neuropsychological Assessment: A Clinical Manual*.

[37] Benton AL, Hamsher KD, Varney NR, Spreen O (1994). *Facial Recognition Test*.

[38] Friedman JH, Friedman H (2001). Fatigue in Parkinson’s disease: a nine-year follow-up. *Movement Disorders*.

[39] Barone P, Antonini A, Colosimo C (2009). The PRIAMO study: a multicenter assessment of nonmotor symptoms and their impact on quality of life in Parkinson’s disease. *Movement Disorders*.

[40] Leyfer OT, Ruberg JL, Woodruff-Borden J (2006). Examination of the utility of the Beck Anxiety Inventory and its factors as a screener for anxiety disorders. *Journal of Anxiety Disorders*.

[41] Abe K, Takanashi M, Yanagihara T (2000). Fatigue in patients with Parkinson’s disease. *Behavioural Neurology*.

[42] Lou JS (2009). Physical and mental fatigue in parkinsons disease: epidemiology, pathophysiology and treatment. *Drugs and Aging*.

